# Cognitive therapeutic exercise in early proprioception recovery after knee osteoarthritis surgery

**DOI:** 10.3389/fresc.2022.915010

**Published:** 2022-08-15

**Authors:** Yubao Ma, Zhijiao Fan, Weiguang Gao, Zihan Yu, Muchen Ren, Quansheng Ma, Dejun Song, Lihua Zhang, Lixin Mi

**Affiliations:** ^1^Musculoskeletal Rehabilitation Center of Beijing Rehabilitation Hospital, Capital Medical University, Beijing, China; ^2^Rehabilitation Treatment Center of Beijing Rehabilitation Hospital, Capital Medical University, Beijing, China; ^3^Department of Graduate, Shenyang Sport University, Shenyang, China; ^4^Nebraska Medical Center, University of Nebraska Medical Center, Omaha, NE, United States

**Keywords:** cognitive therapeutic exercise, positional sensation, knee osteoarthritis, recovery, proprioception

## Abstract

**Objective:**

This research aims to explore the therapeutic effect of cognitive therapeutic exercise (CTE) in proprioception recovery after knee osteoarthritis (KOA) surgery.

**Methods:**

In total, thirty-seven patients recovering from KOA surgery (including 27 patients who had undergone high-tibial osteotomy (HTO) procedure and 10 patients who had received total knee arthroplasty (TKA) treatment were randomly assigned to two groups: 18 patients in the CTE group and 19 patients for the control group (non-CTE). Patients in the CTE group received proprioceptive training as cognitive therapy to facilitate proprioception recovery for up to 4 weeks: 5 days a week and two 10-min sessions a day. Except for cognitive therapeutic exercise, the NCTE group and CTE group had the same treatment protocols. All the interventions began with permission from the surgeon-in-charge. In this research, we applied the joint repositioning training or joint-matching tasks, which is part of the proprioceptive training as a measurement for a proprioceptive training result where patients moved their knee joint from 0° (completely straight knee joint) to produce a presented joint angle, such as 30, 60, and 90° of flexion. Joint-matching task results were recorded before the treatment, at 2 and 4 weeks, postoperatively. The absolute difference between the results of these exercises and the knee flexion angle targets will be measured at each test—pre-rehabilitation (Pre-Reha), 2 weeks post-rehabilitation (2 weeks post-Reha), and 4 weeks post-rehabilitation (4 weeks post-Reha).

**Results:**

The absolute difference in the CTE group was significantly smaller than that of the control group after 4 weeks of treatment (*P* < 0.05). After 2 weeks of cognitive therapeutic exercise, the absolute difference between patients' exercises of joint repositioning and the target angle of 30° in the CTE group was smaller than that of the NCTE group (*P* < 0.01). After 4 weeks of therapy, the joint position sense (JPS) among patients who received cognitive therapeutic exercise when performing joint repositioning at angles of 30 and 60° were better improved than those without receiving proprioceptive training with the absolute difference smaller than those of the control group (*P* < 0.05).

**Conclusion:**

The joint reposition training provided for the CTE group is a painless proprioceptive training practice. This method is simple and effective, making it easy for patients to understand the purpose of training and improve patient engagement. The research showed that after 4 weeks of rehabilitation and physical training, the proprioception sense of both the NCTE and CTE groups improved significantly, and the efficacy of proprioceptive training in the CTE group was better than that of the NCTE group, which provided a new approach to the early proprioception recovery of a patient with KOA after surgery.

## Background

Osteoarthritis of the knee is a common orthopedic disease among the elderly. With a growing aging population in China, the number of elderly patients that could develop knee osteoarthritis is growing accordingly (1–3). Patients with mild symptoms of knee osteoarthritis could choose conservative treatment, namely, taking medications and drugs, cortisone shots and injections, and physical therapy. Surgical treatment is available for patients with symptomatic moderate and severe knee osteoarthritis, namely, unicompartmental knee arthroplasty (UKA), high-tibial osteotomy (HTO), and total knee arthroplasty (TKA), among which HTO and TKA are the most common surgical options for patients with osteoarthritis of the knee (4, 5). The timing of rehabilitation intervention for patient recovery after surgery is very important. With options available and time permitting, the sooner the intervention begins, the better for patient recovery and rehabilitation, especially for future recovery of motor and physical functions among patients. Patients with KOA after surgical treatment should receive systematic rehabilitation treatment to ensure the maximum recovery of their physical functions. At present, post-operative physical therapies and treatments for patients with KOA from home and abroad mainly include a joint range of motion therapy, muscle strength training, proprioceptive training, gait training, physiotherapy, etc. (6–8), which are adjusted based on the conditions of the patients. Apart from proprioceptive therapy, other rehabilitation programs are time-tested and well-developed. (9, 10) In recent years, proprioception therapy for patients after knee osteoarthritis surgical treatment are increasingly popular as proprioceptive deficits undermine the recovery of patients' joint physical functions and mobility (11, 12). Despite the availability of proprioception therapies, there are differences in terms of therapeutic benefits and there is no consensus on the definitive benefits of such therapy.

Cognitive therapeutic exercise, commonly known as the Perfetti Method, is a rehabilitation method developed by the Italian neurologist Carlo Perfetti in the early 1970s and now it is proven effective in postoperative therapy and has been widely used in many countries successfully (13–15). The joint repositioning training in CTE is basically a painless proprioceptive training method. The method is simple and effective and has been applied in the rehabilitation of the neurological diseases and the effect is relatively prominent (16–18), but its application in orthopedic rehabilitation is uncommon. The purpose of this study is to analyze and evaluate the efficacy of CTE therapy in the recovery of proprioceptive sensation of the knee joint after KOA and lay a foundation for the application of CTE in the field of orthopedic rehabilitation in the future. The research hypothesized that proprioception of the knee in both the CTE group and the control group (non-CTE) could be improved after undergoing surgery with better results in proprioceptive function recovery among patients in the CTE group than that of the non-CTE group.

## Methods

### Inclusion and exclusion criteria

Inclusion criteria includes the following: (1) Patients without any cognitive impairment and those who can communicate with others normally; (2) Patients who are eligible for rehabilitation intervention 1 week after KOA surgery; (3) Patients who can actively cooperate and are willing to complete the 4-week rehabilitation program; (4) Patients without other orthopedic diseases or previous history of other conditions on their lower limbs.

Exclusion criteria: (1) Patients with cognitive impairment and are unable to communicate with others normally; (2) Patients with other orthopedic diseases, motor injuries, and nerve injuries of lower limbs; (3) Patients with mental illness; (4) Patients who chose TKA treatment for knee damage that is caused by injury and trauma; (5) Patients with trunk injury or disease affecting the lower limbs.

### The general information

**Figure 1:**
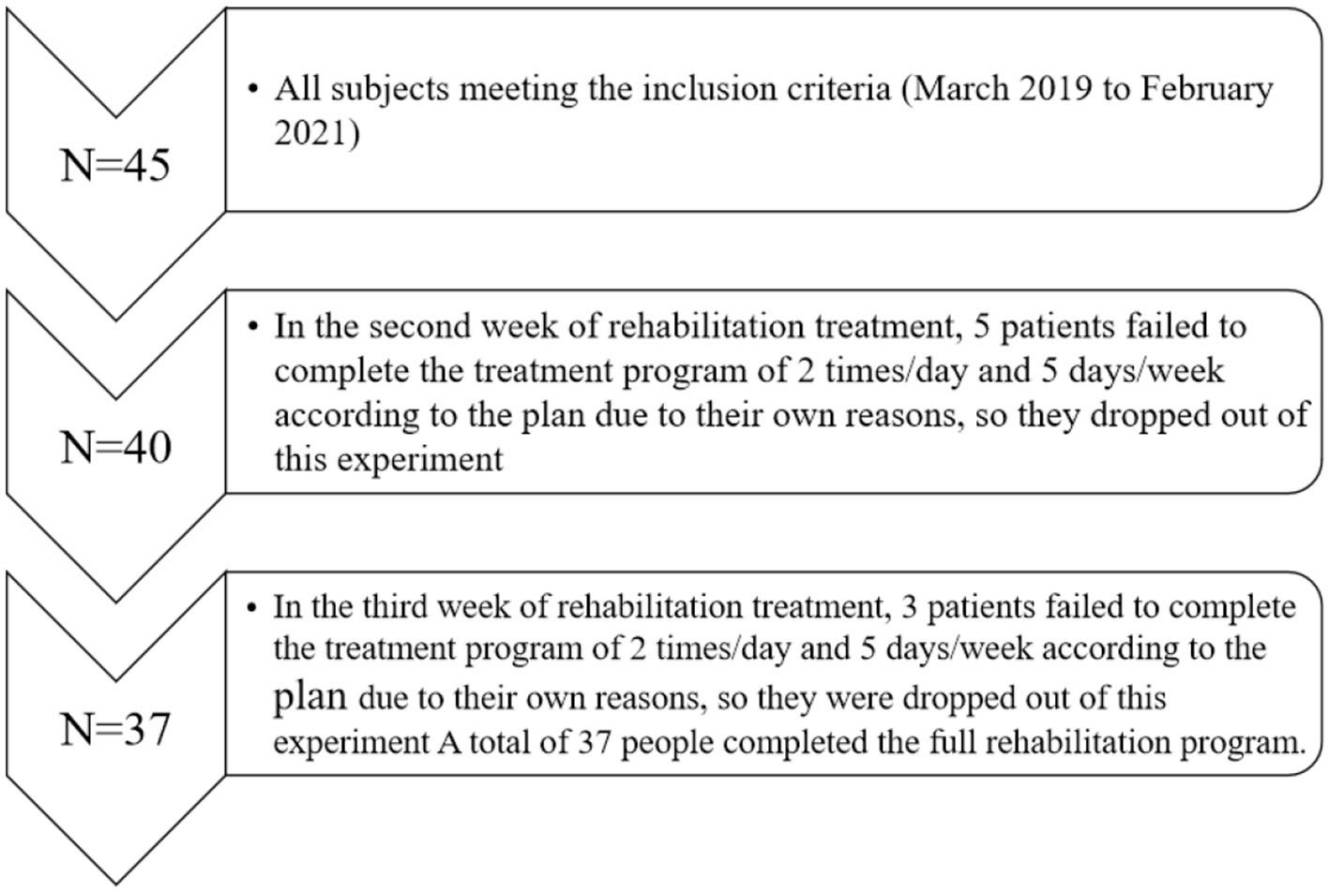
Flow chart of patients participating in this study.

Initially, 45 participants who met the aforementioned inclusion criteria between March 2019 and February 2021 had been recruited for a 4-week rehabilitation program. Among which, eight participants opted out of the program because of personal reasons. A total of 37 patients were able to complete the program and finished all of the tests required (Figure 1) including 27 patients who underwent HTO procedure (of 11 patients with surgery on their left knee) and 10 patients who received TKA surgical treatment (of 6 patients with surgery on their left knee). In total, 37 research participants were randomly divided into two groups: the CTE group (*n* = 18) and the non-CTE group (*n* = 19), and all of whom have an average natural history of such condition for over 4 years (details of patient's information before surgery shown in Table 1). The flow chart of patients' participation and procedure of this experiment was shown in Figure 1. The opt-out by eight patients did not affect the study results. This research has been approved by the Ethics Committee of Beijing Rehabilitation Hospital, Capital Medical University (registration number: 2020BKKY-037), and all the participants have been informed and given their consent to this study.

**Table 1:** Preoperative basic information of patients.

	***n***	**Man/woman**	**Operation side L/R**	**Age(y)**	**Height(cm)**	**Weight(kg)**	**Course of disease(m)**
CTE	18	6/12	9/9	67 ± 4.1	162 ± 3.5	56 ± 4.7	51 ± 4.7
NCTE	19	5/14	8/11	66 ± 2.8	164 ± 2.7	58 ± 3.9	53 ± 2.9
*t*/*x*^2^		2.432	2.856	2.432	1.475	1.385	1.254
*P*	0.352	0.247	0.316	0.663	0.718	0.852	0.843

Values are presented as mean ± SD. CTE, cognitive therapeutic exercise; NCTE, no cognitive therapeutic exercise.

### Rehabilitation treatment

**Figure 2:**
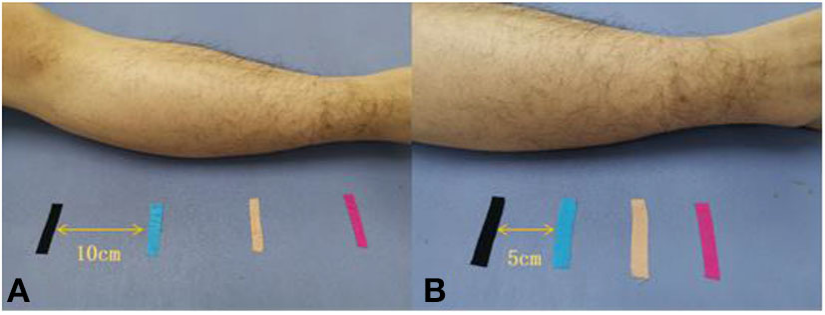
Different color sticky patches were used to fix the marker, **(A)**: each sticky patch is 10 cm apart, **(B)**: each sticky patch is 5 cm apart.

The early rehabilitation program for the non-CTE group mainly included a range of motion training, muscle strength training, proprioceptive training, weight-bearing, and walking training, and other physical therapy for a total of 40 min while the rehabilitation program for the CTE group was slightly different with 30-min standard physical therapy same as the NCTE group and 10-min joint position sense training. The patients were required in the supine position and four markers (adhesive tape with different colors, see Figure 2) were placed on the side of their leg where surgery was performed. The main feature and advantage of CTE are that it always interacts with the patient and patients are paying attention to their body in a conscious way, they are addressing their attention on the position of the foot in space and on the kinesthetic sensations of the knee joint, which not only increases the patient's initiative to participate in the process, improves the degree of cooperation, but also enhances the trust between the therapist and the patient, and provides certain help for a faster and better recovery. What improves the quality of the recovery is the fact that the patient has to feel the body consciously and by being more aware of it, motor function can arise in an easier and more precise way.

**Figure 3:**
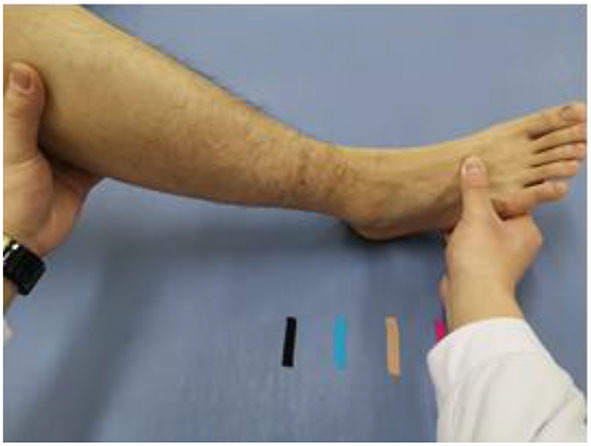
Completely passive stage: the therapist holds one hand on the forefoot of the operative side limb and the other hand on the knee fossa to assist the patient to complete the training movements.

Prior to the therapy program, the specific treatment method of CTE is explained to the participating patient. The treatment is initiated when patients fully understood the procedure and consent to the program. The first training phase (1st week) is a stage when patients are completely passive where the therapist holds one hand on the forefoot of the side with surgery and another hand on the popliteal fossa of the patient to assist patients in completing the required movements for the training (Figure 3). During the treatment, the patient has been in a state of relaxation but at the same time aware and focused on body sensations. Voice prompts on the location of the exercise will be given to patients as the therapist proceed with the physical therapy and repeat the movement for 10 min. To increase the accuracy of such a regimen and boost patients' confidence in their recovery, the designated distance between the markers is set at 10 cm in the initial training, and the subsequent training could gradually reduce the distance between the markers. Patients may be highly aggressive at this stage, so we need to adopt a completely passive approach to training to minimize the pain generated during the session.

The second phase (2nd week) is an active-assisted movement exercise where a therapist holds the heel of the limb that has undergone surgery with one hand and places the other hand at the popliteal fossa of the patient to assist the patient in completing the training movements. During the treatment, the patient is required to keep his eyes closed. When the patient's joint is actively approaching the target angle, the therapist assists the patient in achieving the target angle and lets the patient sense the position of his or her joint at the moment. The movement exercise will be repeated for 10 min. At this stage, the challenging level of the physical training will be increased based on the conditions of the patients, and the distance between markers will also be reduced accordingly.

**Figure 4:**
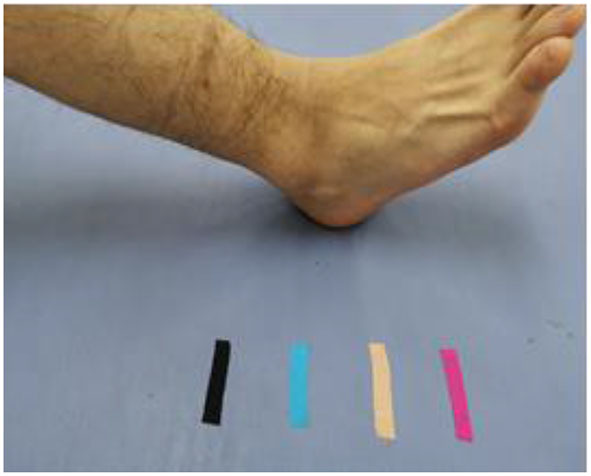
Active movement stage: the treatment process is completely dominated by the patients themselves, and the difficulty of training is gradually increasing.

The third training phase (3rd−4th weeks) is the active movement exercise stage (Figure 4) where the treatment is completely dominated by the patients themselves, and the level of difficulty in physical mobility training is gradually increasing with distances between two markers reduced from initial 10–5 cm. During this, the therapist will give certain verbal reminders and guidance according to the actual situation of the patients. The commonly-used voice prompt at such stage is “Please put your heel at the spot where the pink marker pink is located“, ”Please put your heel at the spot where the beige marker lies“, and so on. The therapist will offer additional prompts when patients fail to move their limbs at the designated location. Patients are required to repeat such exercise for 10 min.

### Joint position sense measurement

We use the Biodex System 4 (Model 850-230, USA-made), a multi-mode computerized robotic dynamometer, where a variable setting device is controlled by a motor that allows the rotation speed of the moving arm to be set at a minimum of 1°/s. When measuring the patient's position, the patient's popliteal fossa should not be in touch with the seat surface by using airbags to block information transmission from the external forces to the foot, which will be fastened to the pedal with a belt.

Patients are required to use eye patches in the process to block out visual information and use headphones with white noise to block out auditory information. The participants hand holds the stop switch and all the commands are transmitted through a microphone headset. According to Borsa et al. (19), the measurement angle is 30, 60, and 90°, and the motion direction is from 0° extension to flexion. Subjects were given the command if you sense that your leg is moving to the prescribed angle, please press the stop button. All the movement angles of the patients will be recorded after they press the stop button. Joint positioning task results are recorded before the therapeutic treatment, at 2 and 4 weeks postoperatively. The absolute difference between the results of these exercises and the knee flexion angle targets will be measured at each test—pre-rehabilitation (Pre-Reha), 2 weeks post-rehabilitation (2 weeks post-Reha), and 4 weeks post-rehabilitation (4 weeks post-Reha).

### Statistical analysis

All the statistical analyses are performed using SPSS Statistics version 22.0, a statistical software suite developed by IBM for data management, advanced analytics, multivariate analysis, business intelligence, and criminal investigation. The statistical test shown that all data conform to a normal distribution, and the results are expressed as mean ± SD. The indexes of the CTE group and NCTE group before treatment, 2 weeks after treatment, and 4 weeks after treatment were statistically analyzed by 2 ways of repeated measures of ANOVA statistical analysis. The level of significance was set at *P* < 0.05.

## Results

**Figure 5:**
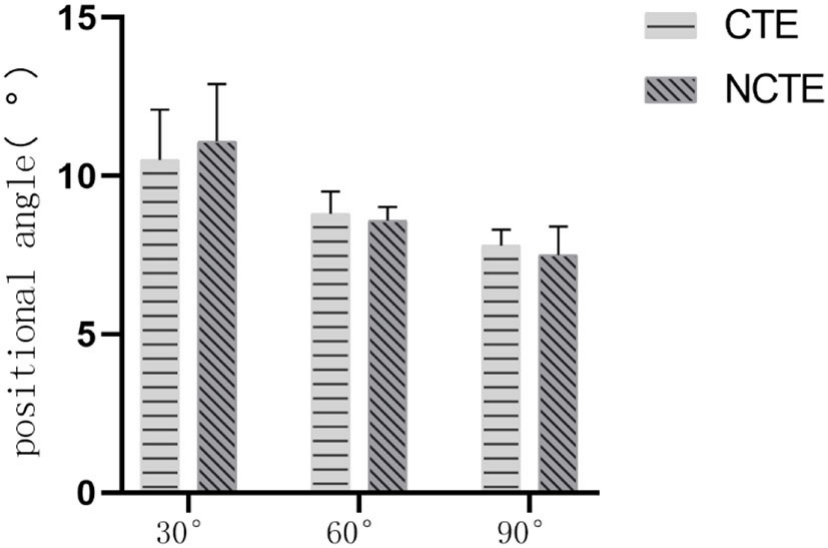
Proprioception before treatment: Before treatment, there was no statistically significant difference in the positional angle between the CTE group and the NCTE group (*P* > 0.05). CTE, cognitive therapeutic exercise; NCTE, no cognitive therapeutic exercise.

**Figure 6:**
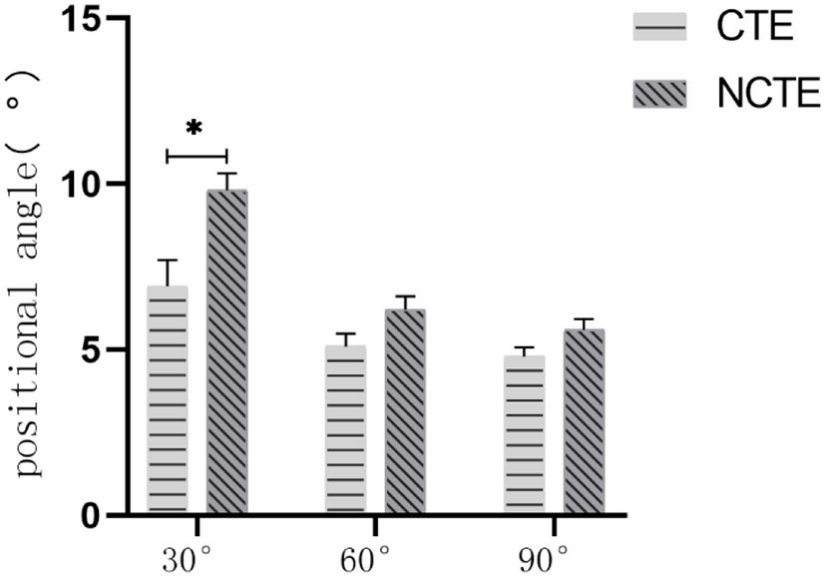
Proprioception after 2 weeks of treatment: At the end of the second week, the angle of the CTE group was smaller than the control group at 30° (*P* = 0.003). There was no statistically significant difference in the positional angle between the two groups at 60 and 90° (*P* > 0.05). CTE, cognitive therapeutic exercise; NCTE, no cognitive therapeutic exercise. *indicates a significant difference between the two groups when completing the position perception test with a target angle of 30°.

**Figure 7:**
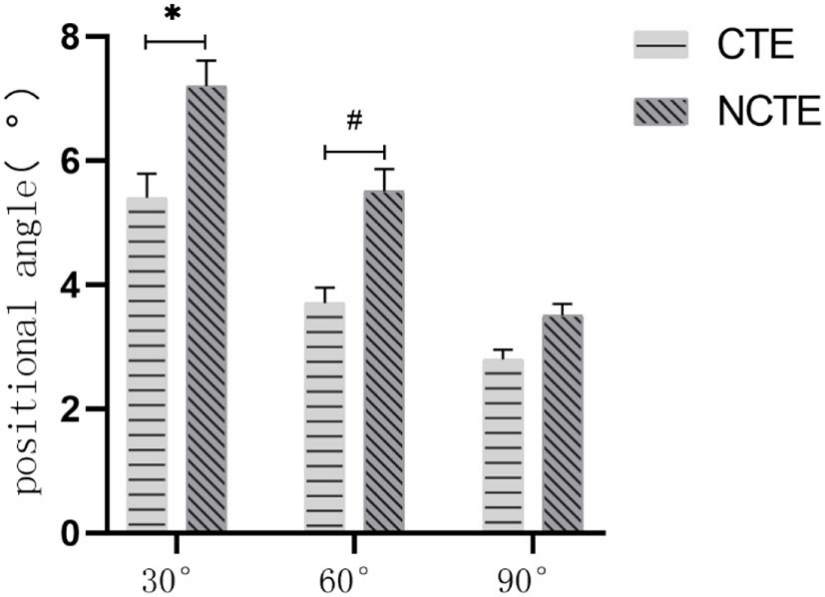
Proprioception after 4 weeks of treatment: At the end of the 4th week, the angle of the CTE group was smaller than the control group at the 30° (*P* = 0.035) and 60° (*P* = 0.042). There was no statistically significant difference in the positional angle between the two groups at 90° (*P* > 0.05). CTE, cognitive therapeutic exercise; NCTE, no cognitive therapeutic exercise. *indicates a significant difference between the two groups when completing the position perception test with a target angle of 30°. # indicates a significant difference between the two groups when completing the position perception test with a target angle of 60°.

Before the physical therapy, there was no statistically significant difference in the joint positioning angle between the CTE group and the NCTE group (Figure 5). After receiving therapy for 2 and 4 weeks, the absolute difference between patients' joint positioning angle and the target angle of the knee was smaller than the value recorded before the treatment in both groups (*P* < 0.05). Approaching the end of the second week of therapy, the absolute difference between the joint positioning angle registered among patients in the CTE group and the target angle was smaller than that of the control group at 30° (*P* = 0.003). There was no statistically significant difference in the absolute difference between the data registered at 60 and 90° of knee flexion (Figure 6). After 4 weeks of therapy, the joint position sense (JPS) among patients who received cognitive therapeutic exercise when performing joint repositioning at angles of 30 and 60° were better improved than those without receiving proprioceptive training with the absolute difference smaller than those of the control group with 30° (*P* = 0.350) and 60° (*P* = 0.042). There was no statistically significant difference between the two groups when the knee bends at 90° (Figure 7).

## Discussion

The position sense training of CTE is a method that patients actively participate and that is not only a process of thinking, but that the patients have to solve a cognitive problem regarding their bodies so they have to look for sensations, by feeling their body and by interpreting what they are feeling with the brain, which has a very positive role in promoting the mobilization of cognitive function of the brain. The training session is divided into three phases: passive movement stage, active-assisted movement stage, and fully active movement stage, and the level of difficulty of the training is gradually increased.

In the passive movement stage, the patient is in a state of complete relaxation with eyes shut. Although the lower limbs do not actively participate in the movement, the brain is fully engaged and functioning. With the therapist's prompt, patients are constantly thinking about the position of their knee joint to enhance the brain's perceptual memory. The level of the exercise and the repetition of movement is constantly adjusted on the basis of the conditions and progress the patients have shown in their physical therapy. In the stage of the active-assisted movement, the patient's active movement is required when considering the progress, they have achieved in the previous session by requiring patients to sense their joint position and movement and move their lower limb. To improve the accuracy of patients' brain memory, the therapist will give certain reminders during the training, which further strengthens the brain's perceptual memory. In the fully active movement stage, patients were fully involved in the exercise on the basis of the previous sessions and patients completed the training content independently at this stage. If necessary, the therapist will give the patient a reminder to remember the exact location of the knee joint, reinforcing the brain's memory of the perception.

Kaas et al. (20) have shown that peripheral lesions at the sensory cortical level that identify early afferent damage are silent and completely non-excitatory meaning from a functional point of view. Therefore, the peripheral lesions remain silent and are often occupied by many incoming projections from regions different from the original area, which are not always equal and do not always exert the same effect.

In humans, abnormal sensory cortical potentials have been demonstrated because of joint degeneration, injury, or disease. Deficiency of afferent nerves from the periphery or changes in processing at higher levels (such as the cortex) only disrupts the organization of various activities, making it difficult to construct the necessary information. Therefore, it is clear that the behavioral activities associated with them can reduce the possibility of variability and increase adaptability only by using compensation strategies and activating them in the right way. The presence of compensation strategies and appropriate motor behavior may improve its function, while inappropriate behavior may increase the variation in data observed. For example, in this study, the recovery effect of proprioception in the CTE group is significant, however, further research is warranted to further investigate in such field, namely, a larger sample size, especially factoring in the correlation between the CTE and other parameters. The destruction of the structural information gathered by sensory neurons can also affect knee function, including all direct effects on the motor neurons near the ligaments or altering the same muscle group to determine atrophy or activation of local strategies. Spencer et al. showed that increased pressure through the afferent pathway of the knee capsule resulted in the quadriceps femoris muscle suppression of motor neurons (21). In turn, muscle activation changes can lead to a lack of disjunctive movement. This may explain the faster recovery of flexion and extension function in the CTE group compared with the NCTE group, and thus, the faster recovery of proprioception. It was assumed that both the CTE group and the NCTE group could improve the knee proprioception of patients after KOA surgery, and the proprioception recovery of the CTE group after treatment was better than the NCTE group. This study verified that both the CTE group and the NCTE group could improve the knee proprioception of patients after KOA, and the proprioception recovery of the CTE group was better than that of the NCTE group at 30 and 60° after 4 weeks of treatment, but there was no significant difference between the two groups at 90°.

## Conclusion

Thanks to early intervention and rehabilitation program, the proprioception of patients with KOA had recovered; the effect of proprioception recovery among patients in the CTE group was better than that of the NCTE group. Our results highlight that CTE training is a safer, less aggressive, and more acceptable rehabilitation intervention and treatment for KOA postoperative patients. However, our study requires a longer follow-up and a larger sample size to further support the claim. In the future, we are interested in continuing to explore the clinical application of CTE in orthopedics and sports injury rehabilitation.

## Limitations

- The patients with KOA condition that had undergone two surgical treatments TKA and HTO were recruited, which may have an impact on the experiment results. In the future, the inclusion criteria shall be changed to include only patients with one type of surgery.
- Routine and standard rehabilitation training and program need to be implemented based on the conditions of the patients involved, which could not guarantee the same training intensity among patients and could lead to certain errors in data.

## Data availability statement

The raw data supporting the conclusions of this article will be made available by the authors, without undue reservation.

## Ethics statement

The studies involving human participants were reviewed and approved by Ethics Committee of Beijing Rehabilitation Hospital affiliated to Capital Medical University. The patients/participants provided their written informed consent to participate in this study.

## Author contributions

YM, LM, and LZ contributed to conception and design of the study. WG and ZY organized the database. QM and DS performed the statistical analysis. YM and ZF wrote the first draft of the manuscript. WG and MR wrote sections of the manuscript. All authors contributed to manuscript revision, read, and approved the submitted version.

## Funding

This research was funded by the 2019–2021 Special Science and Technology Development Project of Beijing Rehabilitation Hospital, Capital Medical University (#2019-046).

## Conflict of interest

The authors declare that the research was conducted in the absence of any commercial or financial relationships that could be construed as a potential conflict of interest.

## Publisher's note

All claims expressed in this article are solely those of the authors and do not necessarily represent those of their affiliated organizations, or those of the publisher, the editors and the reviewers. Any product that may be evaluated in this article, or claim that may be made by its manufacturer, is not guaranteed or endorsed by the publisher.
